# MKRN2 knockout causes male infertility through decreasing STAT1, SIX4, and TNC expression

**DOI:** 10.3389/fendo.2023.1138096

**Published:** 2023-03-10

**Authors:** Lin Wang, Yan-Ling Yong, Kun-Kun Wang, Yun-Xia Xie, Ying-Chen Qian, Feng-Mei Zhou, Jian-Ge Qiu, Bing-Hua Jiang

**Affiliations:** ^1^ The First Affiliated Hospital of Zhengzhou University, Academy of Medical Science, Zhengzhou University, Zhengzhou, Henan, China; ^2^ The Affiliated Jiangning Hospital of Nanjing Medical University, Nanjing, Jiangsu, China

**Keywords:** MKRN2, STAT1, SIX4, TNC, male infertility

## Abstract

*Makorin-2* (*Mkrn2*) is an evolutionarily conserved gene whose biological functions are not fully known. Although recent studies have shed insights on the potential causes of male infertility, its underlining mechanisms still remain to be elucidated. We developed a *Mrkn2* knockout mice model to study this gene and found that deletion of *Mkrn2* in mice led to male infertility. Interestingly, the expression level of signal transducer and activator of the transcription (STAT)1 was significantly decreased in MKRN2 knockout testis and MEF cells. Co-IP assay showed an interaction between MKRN2 and STAT1. Moreover, our results further indicated that MKRN2 regulated the expression level of SIX4 and tenascin C (TNC) *via* the EBF transcription factor 2 (EBF2) in mice. The results of our study will provide insights into a new mechanism of male infertility.

## Introduction

Makorin-2 (MKRN2; HSPC070) belongs to the *MKRN* gene family with the ribonucleoproteins characterized by a variety of zinc-finger motifs, which contains four C3H zinc fingers and a C3HC4 really interesting new gene (RING) zinc finger domain (1, 2). MKRN2 was first identified in human CD34+stem/progenitor cells, as well as in some leukemic cell lines (1, 3, 4). Previous studies reported that mkrn2 in *Xenopus laevis* acted upstream of glycogen synthase kinase-3b in the phosphatidylinositol 3-kinase/Akt pathway. The third C3H zinc finger and the RING motif are required for the antineurogenesis activity (5, 6). Recent studies also investigated the role of MKRN2 in tumorigenesis, such as lung cancer and melanoma (7, 8). Though MKRN2 is a highly conserved gene, however, its function remains largely unknown.

The signal transducer and activator of the transcription (STAT) protein family mediate the transcription of several genes, such as cytokine-inducible genes and growth factors (9–12). So far, seven STAT proteins have been identified: STAT1–6 and STAT5b (13, 14). When the receptor bounds to STATs, a conserved tyrosine residue in the C-terminal domain will be phosphorylated by the Janus kinase (JAK), then two STATs assemble through reciprocal phosphotyrosine/SH2 domain interactions leading to dimerization. Once dimerized, STATs are translocated into the nucleus and regulate the transcription of several different genes. Truchet et al. reported that JAK/STAT pathway is functional during early embryonic development, and STAT1 is present in mouse oocytes and in preimplantation embryos (15). STAT1 was reported to be phosphorylated in response to capacitation and the acrosomal reaction (16). Moreover, human samples with varicocele conveyed a significant negative correlation between the phosphorylated levels of STAT1 and sperm head morphological defects (17). Moreover, The *Six4* genes belong to the mammalian homolog of the *Drosophila sine oculis homeobox* (*Six*) family, and studies have reported that SIX4 is required for genital primordium formation and testicular differentiation of male gonads (18). SIX4 and tenascin C (TNC) have been reported to be functioning in a productive system. Thus, we wonder how to evaluate the regulatory mechanism between MKRN2 and STAT1/SIX4/TNC to figure out the novel mechanism of MKRN2 in male infertility.

In this study, we plan to address: (a) the role of MKRN2 in male infertility; (b) whether MKRN2 regulates the expression level of STAT1; and (c) how MKRN2 induces the expression levels of SIX4 and TNC by the transcription factor EBF transcription factor 2 (EBF2). The results of our study will provide insights into a new mechanism of MKRN2 in regulating male infertility.

## Materials and methods

### Generation of Mkrn2-knockout mice

To generate *Mkrn2*-heterozygous and *Mkrn2-*knockout mice, *Mkrn2*-floxed mice have been crossed with transgenic EIIa-cre mice with a C57BL/6 J background as we previously described (19) to obtain mosaic mice with the *Mkrn2* ^+/flox(−)^·EIIa-cre genotype. The resulting mice were crossed with C57BL/6 J to obtain *Mkrn2* heterozygotes. *Mkrn2* knockout mice were generated by sibmating *Mkrn2* heterozygotes. Genomic DNAs isolated from the tails were genotyped with indicated primers by PCR as described (19). All the animals were housed in specific pathogen-free conditions, and all the experiments were approved by the Committee of Laboratory Animal Experimentation of Zhengzhou University.

### Cell culture

*Mkrn2*-WT and *Mkrn2*-KO primary mouse embryonic fibroblasts (MEFs) were derived from 13.5-day embryos and cultured as described (19), and 293T cells were cultured in the DMEM medium supplemented with 10% FBS, penicillin (100 U/ml), and streptomycin (100 μg/ml), and were maintained at 37°C.

### H&E staining

The H&E staining procedure used here is as follows: (a) Deparaffinize all sections in xylene two times for 10 min each. (b) Gradually rehydrate sections with graded alcohol: wash in absolute alcohol two times for 5 min each, then 95% alcohol for 2 min, and 70% alcohol for 2 min. (c) Briefly wash in distilled water. (d) Stain in Harris hematoxylin solution for 8 min, then wash for 5 min under running water. (e) Differentiate in 1% acid alcohol for 30 s, then wash under running water for 1 min. (f) Blue in 0.2% ammonia water for 30 s, then wash under running water for 5 min. (g) Rinse in 95% alcohol at 10 dips. (h) Counterstain in an eosin–phloxine solution for 1 min. (i) Dehydrate with 95% alcohol and wash with absolute alcohol two times at 5 min each. (j) Clear in xylene two times at 5 min each. (k) Lastly, mount with a xylene-based mounting medium.

### Western blot analysis

Cells were harvested, washed with cold PBS twice, and then suspended in 200 μl of cold cell lysed buffer with protease inhibitor. Tissues were homogenized and suspended in 500 μl of cold cell lysed buffer with protease inhibitor. All of the lysates were incubated on ice for 30 min and centrifuged for 10 min at 4°C; the supernatants were collected, and the protein concentration was quantified using the Bradford method (20). The cell lysates were resolved by SDS-PAGE and transferred onto polyvinylidene fluoride (PVDF) membranes. After being incubated in the blocking buffer (5% BSA in TBST) for 2 h at room temperature, the membranes were incubated with primer antibodies overnight at 4°C, washed three times with TBST, and incubated with HRP-conjugated secondary antibodies for 1 h at room temperature, then washed three times more with TBST. The protein–antibody complex was detected by a chemiluminescence detection system *via* an ECL chemiluminescence detection kit.

### Quantitative real-time PCR

Quantitative real-time RT-PCR was used to determine the gene expression levels. RNAs were extracted using a Trizol reagent, reverse transcribed using SYBR Premix Dimer Eraser, and real-time PCR was performed using QuautStudio-5 Real-Time Thermal Cycler (ABI, Los Angeles, CA, USA). The following primer sequences were used for Q-PCR: Six4 forward primer: 5′-CTCCTGTCTCAGTAGCAGCTTC-3′; reverse primer: 5′-GGAACGGTGTATACCACTGCAC-3′; Ebf2 forward primer: 5′-GAGCAAGAAGGCTTGACCCATC-3′; reverse primer: 5′-CCAAACACAACCTGGAGACCATC-3′; Tnc forward primer: 5′-GAGACCTGACACGGAGTATGAG-3′; reverse primer: 5′-CTCCAAGGTGATGCTGTTGTCTG-3′; and GAPDH forward primer: 5′-ATGGGTGTGAACCATGAGAAGTATG-3′; reverse primer: 5′-GGTGCAGGAGGCATTGCT-3′. The gene expression levels were normalized to the value of GAPDH, and fold changes were calculated by relative quantification (2^−ΔΔCt^).

### Co-immunoprecipitation

HEK293T cells were cultured in a 10-cm plate and transfected with the indicated plasmids for 48–72 h. Cells were then collected and lysed in immunoprecipitation (IP) buffer (1 M Tris, 0.5 M EDTA, 5 M NaCl, 100% glycerol, 100% Np40) and treated with antibodies at 4°C overnight. The lysis was then incubated with agarose beads (Roche, Indianapolis, IN, USA) for 4–6 h at 4°C. IP samples were separated by 8%–10% SDS-PAGE gels, and protein bands were detected by the chemiluminescent substrate.

### Protein quantification and differently expressed protein analysis

Total proteins were extracted from *Mkrn2*-WT and *Mkrn2*-KO MEFs, with four biological repetitions in each group. The proteins of different samples were respectively subjected to trypsin enzymatic hydrolysis, and peptide fractionation as described (21). The samples were further analyzed on a Thermo HFX MS (Thermo Fisher Scientific, Waltham, MA, USA) interfaced with an EASY-nLC1200 LC system (Thermo Fisher Scientific). Raw files were searched against the mouse refseq protein database (27,414 proteins, version 04/07/2013) with Proteome Discoverer (Thermo Fisher Scientific, version 1.4). The levels of proteins were estimated with a label-free, intensity-based absolute quantification (iBAQ) approach (22). The iBAQ of each protein was normalized by dividing the sum of all identified proteins. We replaced the extremely small values with 10^−8^. The matrix file integrated with all samples was subjected to different protein expression between *Mkrn2*-WT MEFs and *Mkrn2*-KO MEFs by using the limma package in R 3.6.1 software (23). Different proteins were filtered by |Log2(Fold Change)|>1 and *p*-value < 0.05. Heatmap shows 977 differently expressed proteins.

### Pathway enrichment analysis

The pathway enrichment analysis of differently expressed proteins was performed with the Reactome database, using the ReactomePA package in R software. Default parameters (OrgDb, org.Mm.eg.db; *p*-value cutoff, 0.05; *q*-value cutoff, 0.2; *p*-value adjust method, BH; readable, T) were set for pathway enrichment analysis. Enriched pathways with FDR < 0.2 were considered statistically significant.

### Differently expressed gene analysis

The matrix data from GEO (GSE6872) is an expression profiling array, and the analysis of differently expressed genes was performed by using the limma package in R software (24). The threshold for differently expressed genes is |Log2(Fold Change)| > 1 and *p*-value < 0.05. The volcano plot shows analysis results including upregulated genes (red), downregulated genes (blue), and nonsignificant genes (black).

### Statistical analysis

All results have been obtained from at least three independent experiments, and data were analyzed *via* GraphPad Prism 8 software. The statistical evaluation for data analysis was determined by a *t-*test. The differences were considered to be statistically significant at *p* < 0.05.

## Results

### MKRN2 is related to male spermatogenesis

**Figure 1:**
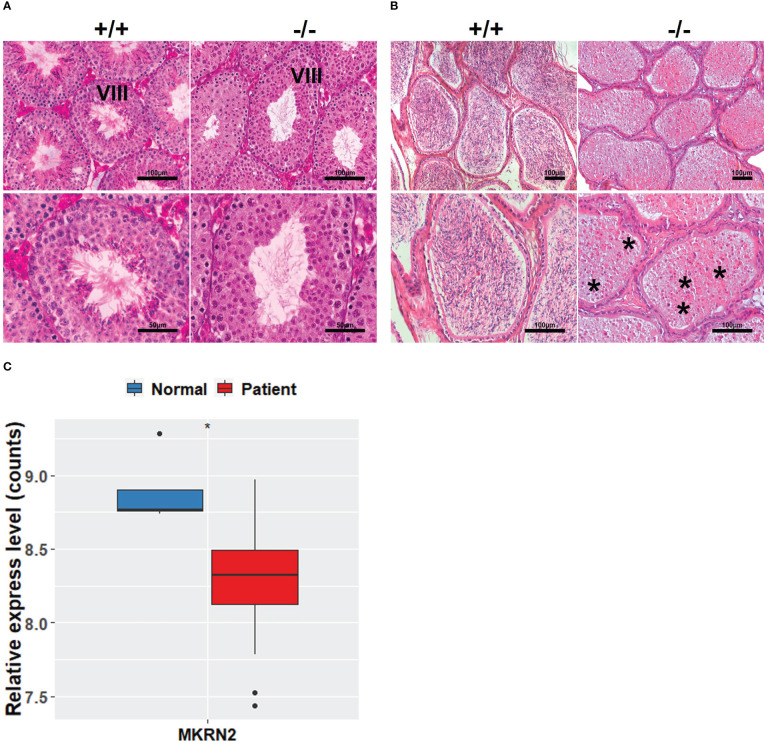
MKRN2 is related to male spermatogenesis. **(A, B)** Hematoxylin and eosin (H&E) staining sections of *Mkrn2*-WT (+/+) and *Mkrn2*-KO (−/−) mice testes in the phase VIII of sperm development and epididymis. Representative pictures are shown, Asterisk represents no sperm. **(C)** Expression levels of MKRN2 in four normal spermatogenesis individuals (normal) and 27 nonobstructive azoospermia samples (patients) from GEO dataset GSE45885. Data are analyzed by two-tailed, unpaired Student’s *t*-test. ^*^ *p* < 0.05, a significant difference.

In order to investigate the effects of MKRN2 on male spermatogenesis, we detected the macroscopic appearance of the testis of *Mkrn2* knockout and wild-type (WT) mice *via* HE staining. We selected the testis at phase VIII of testicular development and epididymis, and the results showed that compared to the wild-type group, there were no sperm in the testis of *Mkrn2* knockout mice (**Figures 1A, B**). In our previous study, we showed that *Mkrn2* knockout induced spermiation failure, but we also found that a few *Mkrn2* knockout mice had another new phenotype in which there is no sperm generated. We then analyzed the MKRN2 expression level of four normal spermatogenesis individuals (normal) and 27 nonobstructive azoospermia samples (patients) from the GEO dataset GSE45885. The results showed that the expression level of MKRN2 was significantly lower than the normal individuals (**Figure 1C**). These results suggested that MKRN2 played a key role in testicular development and male spermatogenesis.

### MKRN2 expression levels are highly correlated with the receptor tyrosine kinase signaling pathway

**Figure 2:**
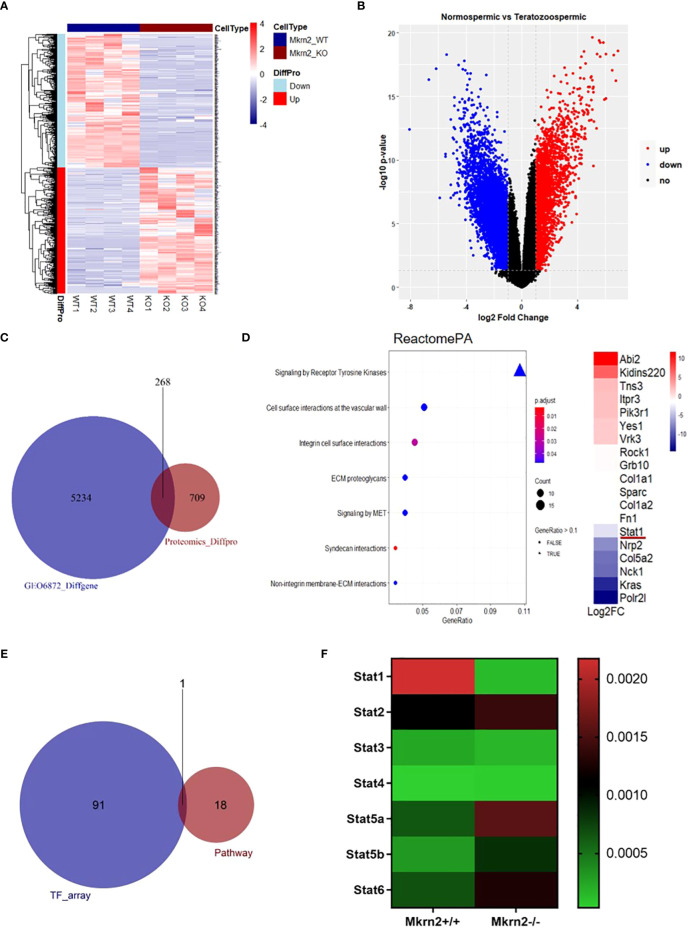
MKRN2 expression levels are highly correlated with the receptor tyrosine kinase signaling pathway. **(A)** Heatmap plot of all differentially expressed proteins in *Mkrn2*-WT MEFs and *Mkrn2*-KO MEFs. The proteins and samples are respectively clustered by Euclidean distance, and the color block shows the classification. Upregulated proteins are coded in red color and downregulated proteins are coded in blue. **(B)** Volcano plot of DEGs in normospermic group and teratozoospermic group from GEO dataset GSE6872. According to the values of logFC and *p*-value (logFC ≥ 1 or ≤ −1, *p* < 0.05), all genes were classified into upregulated genes (red), downregulated genes (blue), and no changed genes (black). **(C)** The overlapping between differentially expressed proteins from proteomics (Proteomics_Diffpro) and DEGs from GSE6872 (GSE6872_Diffgene). **(D)** The signaling pathways are represented among overlapping genes and ranked according to the number of enriched genes. The color of the dots represents the *p*-value; the size of the dot represents the gene count, and the shape of the dots represents the gene ratio. The primary signaling pathway is signaling by receptor tyrosine kinases, including 18 genes, ranked by log2FC. **(E)** The overlapping of the results of the transcription factors array (TF_array) and genes of the signaling by receptor tyrosine kinase pathway. **(F)** Expression levels of the STAT family members in *Mkrn2*-WT MEFs and *Mkrn2*-KO MEFs, according to TF array results.

To investigate the role of MKRN2 in testicular development and male spermatogenesis further, we performed a proteomics analysis of *Mkrn2*-WT MEFs and *Mkrn2*-KO MEFs (**Figure 2A**), as well as an analysis of differential expression genes (DEGs) in normospermic and teratozoospermic groups from the GEO dataset GSE6872 (**Figure 2B**). The signaling pathways represented among overlapping genes and ranked according to the number of enriched genes were screened for overlapping genes between differentially expressed proteins from proteomics and DEGs from GSE6872. The results showed that the primary signaling pathway (**Figures 2C, D**) was receptor tyrosine kinase signaling. We performed a transcription factor assay to compare the different transcription factors between Mkrn2-WT and Mkrn2-KO MEFS, and the overlapping genes of the transcription factor array and genes of the receptor tyrosine kinase signaling pathway were analyzed. STAT1 was the only gene that overlapped (**Figure 2E**). We then analyzed the expression levels of STAT family members in *Mkrn2*-WT MEFs and *Mkrn2*-KO MEFs according to the transcription factor array results, and STAT1 was the most downregulated gene in the *Mkrn2*-KO MEFs (**Figure 2F**). All of these results indicated that MKRN2-regulated STAT1 is involved in the process of male infertility.

### MKRN2 regulates the expression level of STAT1

**Figure 3:**
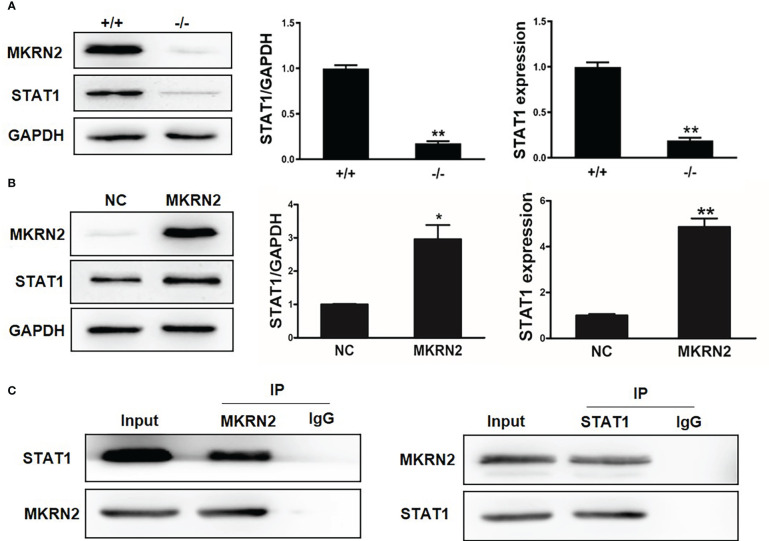
MKRN2 regulates the expression level of STAT1. **(A)** The protein and mRNA expression levels of MKRN2 and STAT1 in *Mkrn2*-WT MEFs and *Mkrn2*-KO MEFs. **(B)** Overexpressed MKRN2 in the 293T cells and the protein and mRNA expression levels of MKRN2 and STAT1 are shown. **(C)** Overexpressed MKRN2 or STAT1 in the 293T cells; the co-immunoprecipitation assay was preformed to detect the interaction of MKRN2 and STAT1, and the representative pictures are shown. ^*^ *p* < 0.05 and ^**^ *p* < 0.01—significant difference.

To further investigate the regulatory effect of MKRN2 on STAT1, we measured the protein and mRNA expression levels of MKRN2 and STAT1 in *Mkrn2*-WT MEFs and *Mkrn2*-KO MEFs, and the results showed that the expression level of STAT1 was significantly downregulated in *Mkrn2*-KO MEFs (**Figure 3A**). We then overexpressed MKRN2 in the 293T cells and measured the protein and mRNA expression levels of STAT1. The results showed that the expression level of STAT1 was significantly upregulated in MKRN2 overexpression cells (**Figure 3B**). We further found that MKRN2 could interact with STAT1 using a co-immunoprecipitation assay (**Figure 3C**). All of these results indicated that MKRN2 could interact with STAT1 and regulate its expression level.

### MKRN2 mediates male teratozoospermia by regulating the expression level of STAT1

**Figure 4:**
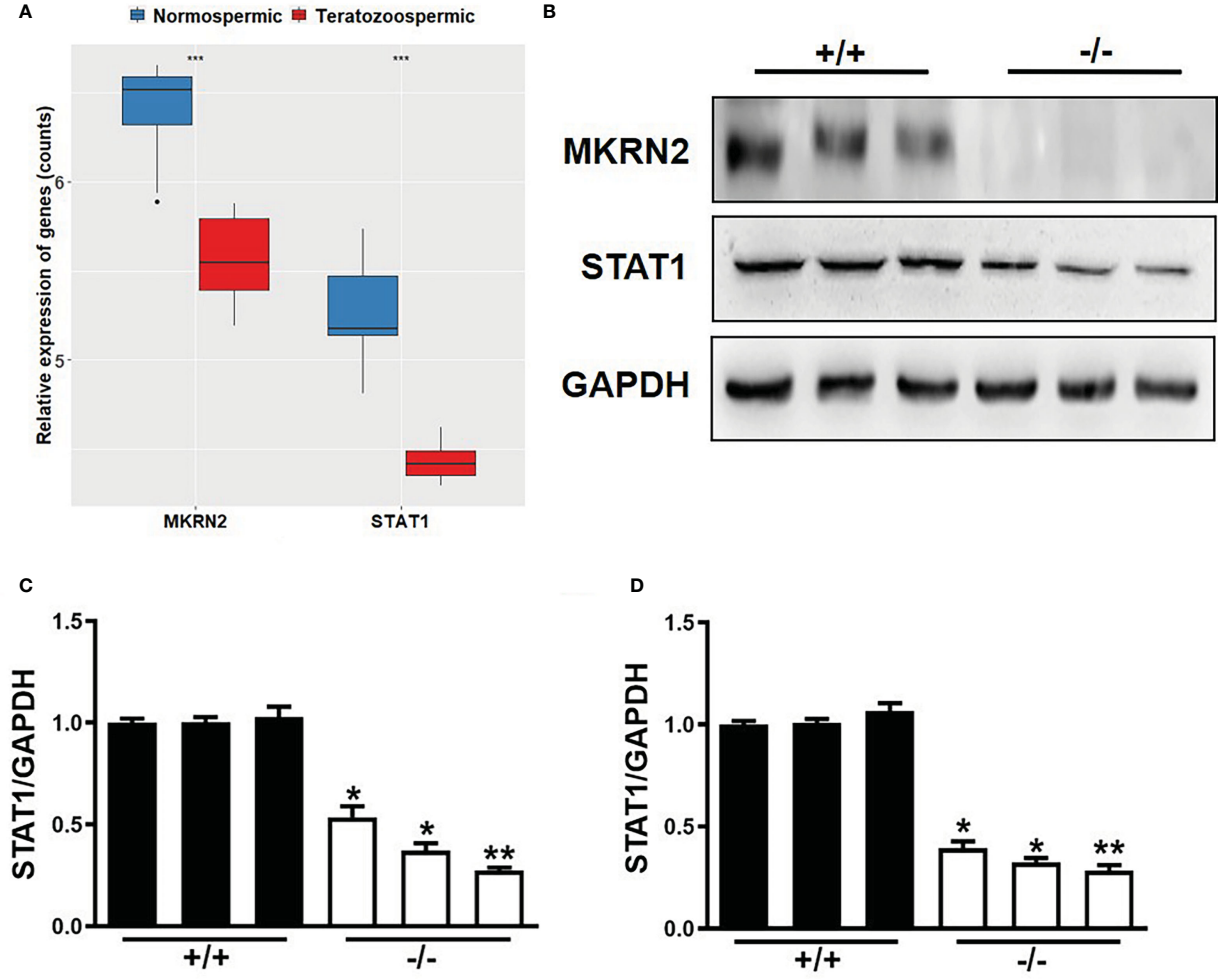
MKRN2 mediates male teratozoospermia by affecting the expression level of STAT1. **(A)** The expression levels of MKRN2 and STAT1 in the sperms of 13 normally fertile male patients and eight infertile individuals with severe and consistent heterogeneous teratozoospermia. Data are from the GEO dataset GSE6872. **(B–D)** The protein and mRNA expression levels of MKRN2 and STAT1 in the testis of *Mkrn2*-WT and *Mkrn2*-KO mice. ^*^ *p* < 0.05, ^**^ *p* < 0.01, and ^***^ *p* < 0.0001—significant difference.

To verify the role of MKRN2 in the male teratozoospermia, we analyzed the expression levels of MKRN2 and STAT1 in 13 normally fertile male patients and eight infertile individuals with a severe and consistent heterogeneous teratozoospermia based on the GEO dataset GSE6872. The results showed that the expression levels of MKRN2 and STAT1 were both significantly downregulated in the infertile patients (**Figure 4A**). We then detected the protein and mRNA expression levels of MKRN2 and STAT1 in the testis of *Mkrn2* knockout and wild-type (WT) mice and found that the expression levels of MKRN2 and STAT1were both significantly downregulated in the testis tissues of *Mkrn2* knockout mice (**Figures 4B–D**). These results indicated that MKRN2 mediated male teratozoospermia by regulating the expression level of STAT1.

### MKRN2 knockout results in the reproductive pathway disorder

**Figure 5:**
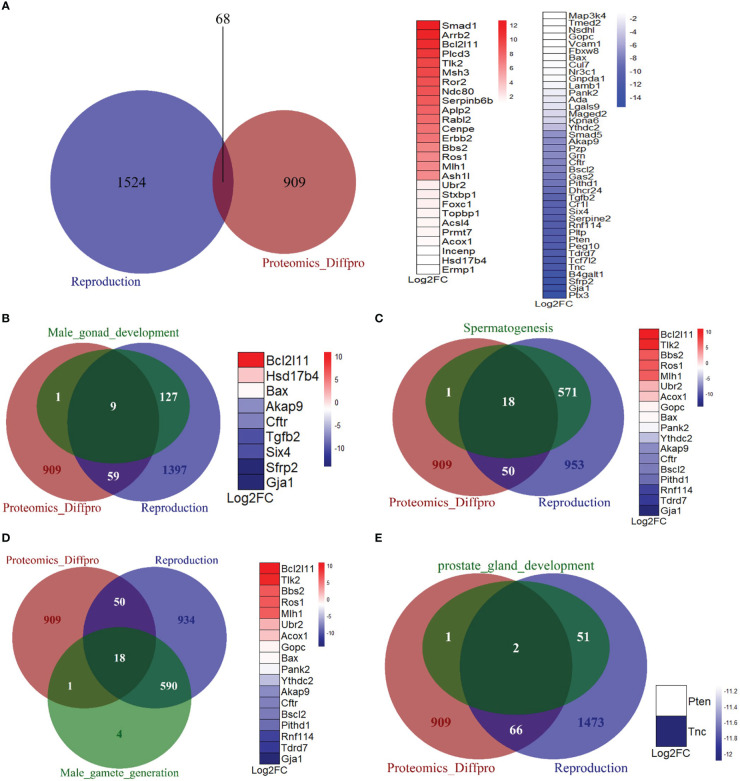
MKRN2 results in a disorder of the reproductive pathway. **(A)** The overlapping between differentially expressed proteins from proteomics (Proteomics_Diffpro) and genes from the reproduction pathway (reproduction). Upregulated proteins (blue) and downregulated proteins (red) from proteomics analysis were ranked by log2FC. **(B–E)** The overlapping of differentially expressed proteins from proteomics (Proteomics_Diffpro), genes from reproduction pathway (reproduction), and genes from related molecular function sets of male infertility, including male gonad development, spermatogenesis, male gamete generation, and prostate gland development. The overlapped genes are ranked by log2 fold change (Log2FC).

To further investigate the effects of Mkrn2 on other reproductive pathways, we obtained a dataset of all genes related to reproduction from the Mouse Genome Informatics (MGI) website (http://www.informatics.jax.org/), overlapped the differentially expressed proteins of *Mkrn2*-WT MEFs and *Mkrn2*-KO MEFs and the reproduction-related genes, and then obtained 68 overlapped genes (**Figure 5A**). We then looked into the overlap of differentially expressed proteins from proteomics, genes from reproduction pathway, and genes from related molecular function sets of male infertility. There were nine overlapping genes with male gonad development (**Figure 5B**), 18 with spermatogenesis (**Figure 5C**), 18 with male gamete generation (**Figure 5D**), and two with prostate gland development (**Figure 5E**). All of the above findings suggested that MKRN2 knockout cause a disruption in the reproductive pathway.

### MKRN2 regulates the expression of SIX4 and TNC

**Figure 6:**
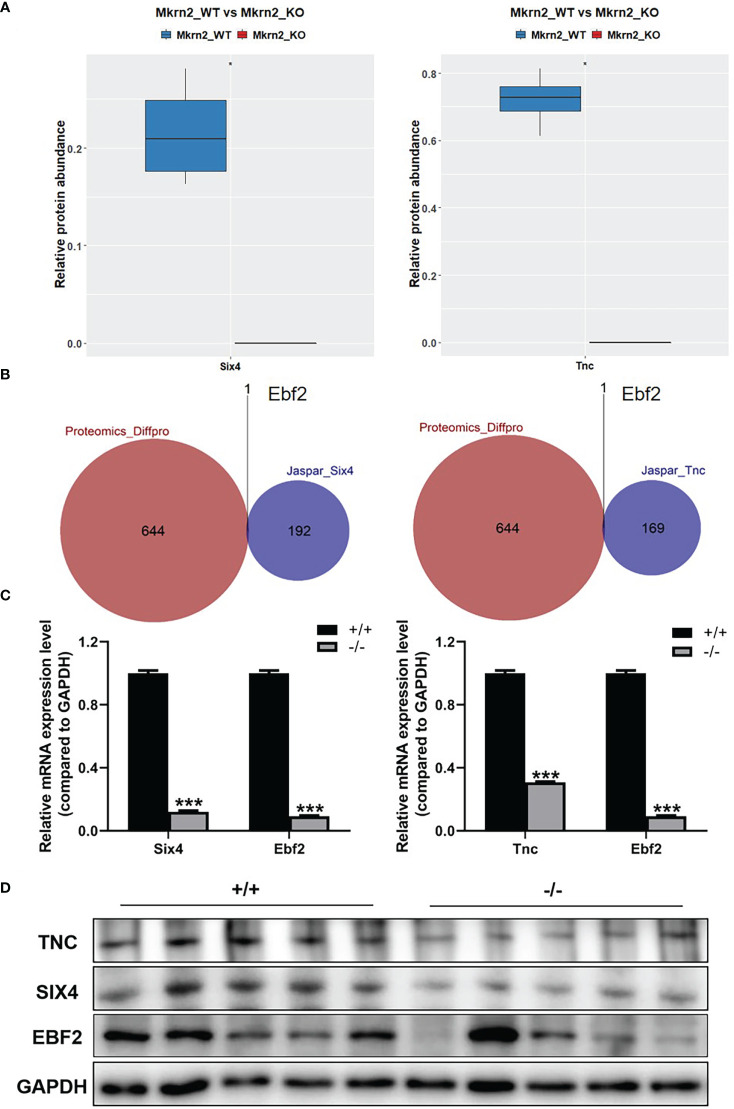
MKRN2 regulates the expression of SIX4 and TNC. **(A)** Proteomics data show SIX4 and TNC expression levels in *Mkrn2*-WT and *Mkrn2*-KO MEFs. **(B)** The overlapping between the putative upstream transcription factors of Six4 or Tnc according to the Jaspar website and differentially expressed proteins from proteomics. Ebf2 is the only differentially expressed transcription factor. **(C)** The mRNA expression levels of Six4 and Ebf2 were detected *via* Q-PCR, and the mRNA expression levels of Tnc and Ebf2 in *Mkrn2*-WT MEFs and *Mkrn2*-KO MEFs were also detected *via* Q-PCR. **(D)** The protein expression levels of EBF2, SIX4, and TNC were detected *via* Western blot in the testis tissues of *Mkrn2*-WT and *Mkrn2*-KO mice. ^*^ *p* < 0.05 and ^***^ *p* < 0.0001—significant difference.

To further investigate the molecular mechanism of the above reproductive-related genes in male teratozoospermia, we verified expression levels of SIX4 and TNC in *Mkrn2*-WT MEFs and *Mkrn2*-KO MEFs using the proteomics data. The results showed that the expression levels of SIX4 and TNC were significantly downregulated in the *Mkrn2*-KO MEFs (**Figure 6A**). We then overlapped the putative upstream transcription factors of SIX4 or TNC according to the Jaspar website and differentially expressed proteins from proteomics and found only one transcription factor, EBF2 (**Figure 6B**). The mRNA expression levels of SIX4, TNC, and EBF2 were detected in *Mkrn2*-WT MEFs and *Mkrn2*-KO MEFs, and we found that the expression levels of SIX4, TNC, and transcription factor EBF2 were significantly downregulated in *Mkrn2*-KO MEFs (**Figure 6C**). We also used Western blot to examine the testis tissues of *Mkrn2*-WT and *Mkrn2*-KO mice, and the results showed that the protein expression levels of EBF2, SIX4, and TNC were decreased in *Mkrn2*-KO mice (**Figure 6D**). These findings indicated that MKRN2 regulated the expression level of SIX4 and TNC in *Mkrn2* knockout mice *via* the transcription factor EBF2.

## Discussion

The *MKRN2* gene is a member of the makorin gene family, which also includes MKRN1, MKRN2, and MKRN3. MKRN2 has been found to be highly conserved throughout evolution, and its ancestral origin can be traced back to 450 million years ago, possibly as a result of *MKRN1* gene duplication (1). We have illustrated the functional role of Mkrn2 in *Xenopus* embryos to negatively regulate neurogenesis through PI3K/Akt signaling. However, the potential functions and molecular mechanisms of MKRN2 in mammals remain to be studied. This *Mkrn2* knockout mouse model and an independent GEO dataset of human sperm samples were used in this study to demonstrate the important role of MKRN2 in male fertility.

Previous studies have reported that MKRN2 inhibits the cell migration and invasion of non-small cell lung cancer by downregulating the PI3K/Akt pathway and is associated with lymph node metastasis, TNM stage, and cell differentiation (7). MKRN2 also acts as an E3 ligase for the NF-κB p65 subunit and has a negative regulatory effect on the inflammatory response (25).

Few reports have focused on the regulation of MKRN2 in male infertility. Our previous results demonstrated that *Mkrn2* deletion in somatic Sertoli cells disrupted ectoplasmic specialization (ES), resulting in abnormalities in sperm heads and spermiation failure, both of which are essential to spermiogenesis. Moreover, *Mkrn2* is crucial for protecting germ cells from apoptosis in spermatogenesis and male fertility *via* the p53/PERP signaling pathway. In this study, we focus on another phenotype of *Mkrn2* knockout mice: no sperm generated, which may indicate the essential role of MKRN2 in male fertility. In this study, we found a new regulatory mechanism between MKRN2 and STAT1/SIX4/TNC, which is a novel mechanism of MKRN2 in regulating male infertility.

Recent studies have shown that the testis shares many similarities with cancerous tissues, including cell division, immigration, and immortalization. Cancer–testis (CT) antigens are usually expressed only in testis tissues, with the exception of early-developing embryos. In addition, CT antigens are expressed in various tumor types (26). The CT genes, OIP5, TAF7L, and AURKC have been identified as biomarkers for breast cancer and may be promising and potent candidates for therapeutic cancer vaccines (27, 28). MKRN2 may function as a cancer–testis antigen, with dual roles in tumorigenesis and spermiogenesis that need to be further investigated.

In this study, MKRN2 could interact with STAT1 and regulate its expression level, as well as the expression levels of SIX4 and TNC *via* the transcription factor EBF2. These results suggested that MKRN2 played a key role in testicular development and male spermatogenesis.

## Data availability statement

The original contributions presented in the study are included in the article/supplementary material. Further inquiries can be directed to the corresponding authors.

## Ethics statement

The animal study was reviewed and approved by Committee of Laboratory Animal Experimentation of Zhengzhou University.

## Author contributions

J-GQ and B-HJ designed experiments and prepared the manuscript. LW, Y-LY, and K-KW performed experiments and analyzed the data. Y-XX, Y-CQ, and F-MZ analyzed clinical samples and provided technical support. B-HJ provided advice. All authors contributed to the article and approved the submitted version.
